# New Particle Formation and Growth from Dimethyl Sulfide Oxidation by Hydroxyl Radicals

**DOI:** 10.1021/acsearthspacechem.0c00333

**Published:** 2021-03-25

**Authors:** Bernadette Rosati, Sigurd Christiansen, Robin Wollesen de Jonge, Pontus Roldin, Mads Mørk Jensen, Kai Wang, Shamjad P. Moosakutty, Ditte Thomsen, Camilla Salomonsen, Noora Hyttinen, Jonas Elm, Anders Feilberg, Marianne Glasius, Merete Bilde

**Affiliations:** † Department of Chemistry, Aarhus University, Langelandsgade 140, Aarhus C DK-8000, Denmark; ‡ Division of Nuclear Physics, Lund University, P.O. Box 118, Lund SE-221 00, Sweden; § Nano and Molecular Systems Research Unit, University of Oulu, P.O. Box 3000, Oulu FI-90014, Finland; ∥ Department of Biological and Chemical Engineering, Aarhus University, Finlandsgade 12, Aarhus N DK-8200, Denmark; ⊥ Faculty of Physics, University of Vienna, Boltzmanngasse 5, Vienna AT-1090, Austria; # Clean Combustion Research Center, King Abdullah University of Science and Technology, Thuwal KSA-23955, Saudi Arabia; ∇ Department of Applied Physics, University of Eastern Finland, P.O. Box 1627, Kuopio FI-70211, Finland

**Keywords:** atmospheric simulation chamber, dimethyl sulfide, methanesulfonic acid, photo-oxidation, nucleation, growth rate

## Abstract

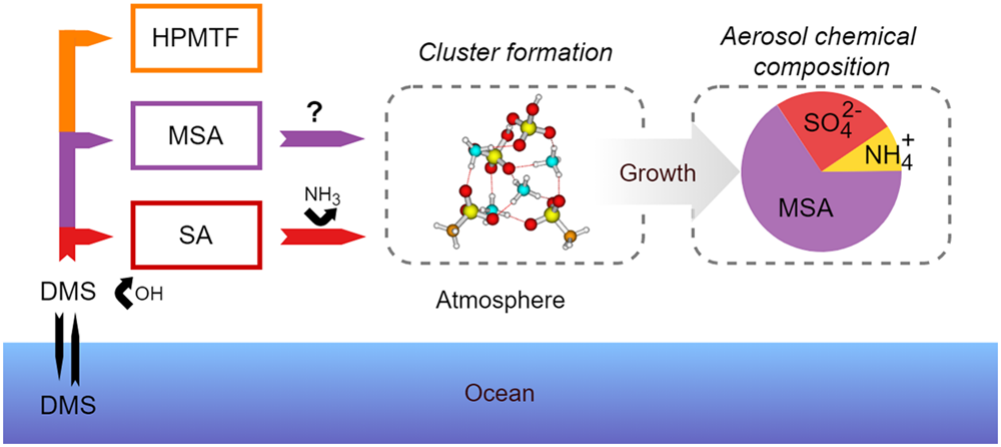

Dimethyl sulfide (DMS) is produced by plankton in oceans and constitutes the largest natural emission of sulfur to the atmosphere. In this work, we examine new particle formation from the primary pathway of oxidation of gas-phase DMS by OH radicals. We particularly focus on particle growth and mass yield as studied experimentally under dry conditions using the atmospheric simulation chamber AURA. Experimentally, we show that aerosol mass yields from oxidation of 50–200 ppb of DMS are low (2–7%) and that particle growth rates (8.2–24.4 nm/h) are comparable with ambient observations. An HR-ToF-AMS was calibrated using methanesulfonic acid (MSA) to account for fragments distributed across both the organic and sulfate fragmentation table. AMS-derived chemical compositions revealed that MSA was always more dominant than sulfate in the secondary aerosols formed. Modeling using the Aerosol Dynamics, gas- and particle-phase chemistry kinetic multilayer model for laboratory CHAMber studies (ADCHAM) indicates that the Master Chemical Mechanism gas-phase chemistry alone underestimates experimentally observed particle formation and that DMS multiphase and autoxidation chemistry is needed to explain observations. Based on quantum chemical calculations, we conclude that particle formation from DMS oxidation in the ambient atmosphere will most likely be driven by mixed sulfuric acid/MSA clusters clustering with both amines and ammonia.

## 1 Introduction

Dimethyl sulfide (DMS) is omnipresent in surface seawater and the marine atmosphere.^1−3^ DMS is believed to be produced by phytoplankton from the decomposition of dimethyl sulfoniopropionate^4^ and constitutes the largest natural emission source of sulfur compounds into the ambient atmosphere.^5^ Once emitted into the atmosphere, DMS can react with atmospheric oxidants with the dominating oxidation process being reactions with the hydroxyl radical (OH). The reaction of DMS with OH radicals can proceed via either an addition pathway or a hydrogen abstraction pathway^6^
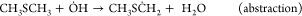
1a
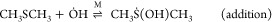
1bThe relative importance of the two pathways at atmospheric pressure depends on temperature. The final product distribution depends on the succeeding chemical reactions influenced by several factors such as temperature, pressure, and NO_*x*_ level.^6,7^ Eventually, the oxidation process leads to the low-volatility acids methanesulfonic acid (CH_3_SO_3_H, MSA) and sulfuric acid (H_2_SO_4_, SA)^6,7^ as well as hydroperoxymethyl thioformate (HOOCH_2_SCHO, HPMTF).^8^

DMS first gained notoriety for its role in the so-called CLAW hypothesis.^9^ This hypothesis presumes a feedback loop where warmer temperatures increase DMS concentrations, leading to more sulfate aerosols and hence more sulfate-containing cloud condensation nuclei (CCN), which increase cloudiness and thereby lead to colder temperatures, potentially mitigating consequences of a warming climate. Although the CLAW concept has been questioned,^10−12^ it has triggered much research in the field; however, it has so far been difficult to prove or disprove the idea.

It is well known that SA is a prime driver of atmospheric new particle formation,^13^ but little is known about the contribution from MSA to new particle formation. The atmospheric concentrations of MSA are usually on the order of 10^5^–10^7^ molecules cm^–3^,^14−20^ which are comparable to typical concentrations of H_2_SO_4._^13^ As anthropogenically emitted SO_2_ is expected to decline in the future,^21−25^ the contribution of MSA to new particle formation is expected to increase.^23^ It is crucial to obtain a better understanding of nucleation and the early growth processes of aerosol particles as these mechanisms govern the formation of cloud condensation nuclei (CCN), which constitute the largest uncertainty in global climate estimations.^26^ For instance, up to half of the formed number of CCN may originate from nucleation^27^ and even minor changes in the parameterization of the early growth of 1.7–3.0 nm particles can lead to a factor of 2 change in the modeled number of CCN.^28^ New particle formation occurs via the formation of stable clusters that subsequently grow to larger sizes.^29^ SA has been shown to cluster efficiently with atmospheric bases such as ammonia^30^ and amines.^31−38^ MSA nucleation has in the past few years been extensively studied using a flow reactor setup, with complementary quantum chemical calculations by the group of Finlayson-Pitts. These studies have shown that water, ammonia, and amines (methyl-, dimethyl-, and trimethylamine) significantly enhance MSA nucleation.^39−45^ The combined effect of MSA and SA on new particle formation has been studied using quantum chemical methods. Bork et al.^46^ showed that at MSA concentrations relevant to the marine environment, cluster formation between SA and dimethylamine could be enhanced from 15 to 300% at 298 and 258 K, respectively, by the presence of MSA. Using a combination of flow tube experiments and quantum chemical calculations, Wen et al.^47^ recently showed that adding MSA to formed SA–water particles led to a bimodal particle distribution with an initial narrow peak that was attributed to MSA–water particles and a broad larger peak that was attributed to SA–water particles. Adding SA to formed MSA–water particles did not show this bimodal behavior.

In this work, we present state-of-the-art chamber experiments targeting the oxidation of DMS with OH radicals under dry, low-NO*_x_*conditions. Using complementary quantum chemical calculations and the Aerosol Dynamics gas- and particle-phase chemistry model for laboratory CHAMber studies (ADCHAM),^48,49^ we seek to elucidate the mechanism for new particle formation from the oxidation of DMS and the role of MSA in particular. Since ammonia is omnipresent in the atmosphere and trace amounts of ammonia seem unavoidable in chamber studies,^30^ our modeling includes the effect of ammonia on new particle formation. A companion manuscript^50^ provides further details on ADCHAM modeling.

## 2 Materials and Methods

### 2.1 Chamber Experiments

All experiments were conducted at the AURA atmospheric simulation chamber, which is described in detail elsewhere.^51^ In short, the AURA chamber facility consists of a 5 m^3^ Teflon FEP bag situated inside a temperature-controlled room. Before each experiment, the chamber was filled with purified dry air (active carbon, HEPA filter, and zero-air generator (Aadco Model 737-14)) at atmospheric pressure and a temperature of 293 K. Nitrogen oxide (NO_*x*_) levels were continuously monitored with a chemiluminescent monitor (AC32M, Environnement S.A.; detection limit, 1 ppb). All experiments were performed at dry conditions (RH < 15%). The chamber has 24 UV lamps (wavelengths 300–400 nm) mounted on the top and bottom of the Teflon bag, and OH radicals were produced by photolysis of H_2_O_2_. The H_2_O_2_ (30% in H_2_O, Merck, 1.07209.0250) was evaporated from a heated round-bottom flask (403 K) and flushed into the chamber using heated N_2_ (333–343 K, 10 L/min). The OH radical concentrations were investigated in separate experiments using the decay of 1-butanol, and results indicate that OH radical concentrations were in the range of typical tropospheric concentrations^52^ (4.64–5.71 × 10^6^ molecules/cm^3^). More details on OH radical estimation can be found in the SI (Table S1, Fig. S1).

In this work, DMS (Sigma-Aldrich, anhydrous ≥99.0%, 274 380) was added to the chamber in four different concentrations ranging from 50 to 400 ppb by transferring a known amount of DMS to a 10 mL glass manifold. DMS was evaporated and flushed into the chamber by a heated N_2_ flow (333–343 K, 10 L/min) to hinder condensation in the tubing during the injection. Table 1 presents an overview of the experimental conditions. In the experiments, the UV lights were turned on first and then H_2_O_2_ was injected followed by DMS. The point in time when DMS was injected was marked time-0 in the analysis. The total instrument sampling rates ranged between 4.2 and 6.9 L/min, resulting in an approximate chamber air residence time of between 12 and 20 h during the experiments.

**Table 1:** Experimental Conditions^a^

exp.	date	DMS	*Υ*	*m*_a_	*N*_tot_	ρ	GR	MSA/SO_4_^2–^
		[ppb]		[μg/m^3^]	[#/cm^3^]	[g/cm^3^]	[nm/h]	
1	05.04.2018	200		28.4	2.2 × 10^7^	1.54	11.7 ± 0.1	3.8
2	19.05.2018	200	0.079	31.8	1.5 × 10^7^	1.56	24.4 ± 2.5	3.0
3	21.05.2018	100	0.044	8.8	1.5 × 10^7^	1.51	10.1 ± 1.2	10.4
4	23.05.2018	50	0.039	4.4	0.5 × 10^7^	1.52		7.1
5	26.05.2018	100	0.051	11.5	0.9 × 10^7^	1.54		4.3
6	27.09.2018	50		2.3	0.4 × 10^7^	1.61	8.8 ± 0.7	1.4
7	15.11.2018	100		5.6	0.9 × 10^7^	1.56	15.1 ± 0.8	3.1
8	19.11.2018	50		3.3	0.9 × 10^7^	1.52	8.2 ± 0.2	6.2
9	13.02.2019	200	0.023	6.5	0.6 × 10^7^	1.55	13.1 ± 0.6	3.2
10	04.03.2019	400		11.3	0.6 × 10^7^	1.62	15.9 ± 1.1	1.3
11	07.03.2019	400		11.2	0.8 × 10^7^	1.51	15.5 ± 1.8	3.2

a RH and temperature in the chamber were <15% and 293 K in all experiments, respectively. In total, 418 μL of H_2_O_2_ were used in Exp. 1–9 and 1500 μL in Exp. 10 and 11. The aerosol mass yield (Υ), maximum aerosol mass (*m*_a_), total number concentration (*N*_tot_), aerosol density (ρ) as calculated from AMS data (see the text), growth rate (GR), and ratio of MSA to SO_4_^2–^ as obtained from AMS measurements.

### 2.2 Particle Size Distributions

Particle size distributions were measured using a TSI scanning mobility particle sizer (SMPS, model 3938). The SMPS system consisted of an electrostatic classifier (model 3082) equipped with either a long (model 3081) or nano (model 3085A) differential mobility analyzer (DMA), an aerosol neutralizer (Kr-85 source, model 3077A) connected to a nano water-based condensation particle counter (WCPC, model 3788). Aerosol instrument manager (AIM) version 10.2.0.11 was used to correct for diffusion losses and multiple charge correction. The combination of the two DMA columns enables measurements of particles in the range 2–422 nm. The SMPS was always measuring with a sheath-to-aerosol flow ratio of 10:1. An impactor (0.071 cm) was installed in front of the DMA, giving a *D*_50_ of 982 nm assuming a density of 1 g/cm^3^.

During the initial nucleation and growth period, only the WCPC was measuring to capture the increase in particle concentration at 1 Hz frequency. After the particle number concentration peaked, the WCPC was connected to the classifier with the nano-DMA column attached (aerosol-to-sheath flow rate of 1.5:15 L/min, size range 2–65 nm). Before the particle size distribution grew out of the size range, the nano-DMA was replaced by the long-DMA column for the remainder of the experiment (aerosol-to-sheath flow rate of 0.6:6 L/min, size range 10–422 nm). The total particle mass was obtained from the SMPS measurement assuming spherical particles, and the density was as retrieved from the HR-ToF-AMS data (see Table 1). The SMPS mass concentration of the polydisperse aerosol particles was wall-loss-corrected following Crump et al.^53^ and Leskinen et al.^54^

### 2.3 DMS Measurements

In five experiments, the concentration of gas-phase DMS in the AURA chamber was monitored by a proton transfer reaction mass spectrometer (PTR-MS, Ionicon Analytik, Innsbruck, Austria^55−57^). PTR-MS is based on chemical ionization of compounds by proton transfer from hydronium (H_3_O^+^) in a drift tube and subsequent detection of ionized compounds using either a high-sensitivity quadrupole (PTR-QMS; Exp. 2–5) or time-of-flight (PTR-ToF 4000; Exp. 9) mass spectrometer. The PTR-QMS was operated under standard conditions with a drift tube voltage of 600 V, a drift tube temperature of 75 °C, and a drift tube pressure of 2.2 mbar with an E/N number of 135 Td. PTR-ToF was run with drift tube conditions of 650 V, 80 °C, and 2.4 mbar and thus an E/N number of 143 Td. DMS (CH_3_SCH_3_) was detected as the protonated molecular ion on a mass-to-charge ratio (*m*/z) of 63. The data was analyzed using PTR-MS Viewer 3 (Ionicon), and a transmission calibration was performed based on specific reaction rate constants and mass discrimination factors prior to the experiments as previously described elsewhere.^58,59^ To minimize sampling losses, the heated inlet was kept at 80 °C and prolonged with a 2 m long Teflon tube, wrapped in heating tape set to 60 °C, and an additional sheath flow of 200 mL/min was chosen. No wall loss corrections were applied to gas-phase data.

### 2.4 Particulate Chemical Composition

A high-resolution time-of-flight aerosol mass spectrometer (HR-ToF-AMS, Aerodyne Research Inc.) was used for real-time analysis of the chemical composition of the total nonrefractory particulate matter. The working principle of the HR-ToF-AMS has been reported in detail elsewhere.^60^ The AMS was connected to the AURA smog chamber by 4 mm ID stainless steel and copper tubing of 1.7 m length (flow rate 0.08 L/min, residence time 15 s) and operated in V-mode in the mass range *m*/*z* of 8–407 with an acquisition cycle of 10 s MS (5 s open, 5 s closed) and 10 s ePToF saved in 1 min averages. AMS data were processed by data analysis software packages SQUIRREL (version 1.61) and PIKA (version 1.21) in Igor Pro. The PIKA default AMS collection efficiency (CE) of 1 was used.

The AMS was calibrated for MSA to achieve accurate quantification and account for its fragments being distributed across both the organic and sulfate species in the default AMS fragmentation table. The calibration was based on the method validated by Hodshire et al.,^61^ i.e., CE = 1, and AMS response to MSA independent of aerosol acidity. The MSA quantification used the specific marker ion CH_3_SO_2_^+^.^62,63^ The calibration setup consisted of an aqueous solution of 4 mM MSA (>99%, Sigma-Aldrich, 471356), an atomizer (TSI 3076), a silica dryer, the above-mentioned SMPS system where the DMA selected dry monodisperse 300 nm particles (RH < 40%), and dilution using particle-free air. The calibration resulted in the ratio of the marker ion to the total MSA signal, *f*(CH_3_SO_2_^+^), equal to 7.35%, and the relative ionization efficiency of MSA, RIE_MSA_, equal to 1.06. The measured mass spectrum of MSA was applied to the HR fragmentation table, as shown in the Supporting Information (Table S2). The AMS data displayed are not corrected for wall losses.

### 2.5 ADCHAM Model Setup

Details on the ADCHAM model setup and DMS chemistry are provided in Wollesen de Jonge et al.^50^ Model parameters specific to the current work, which represent dry conditions only, are given in the SI. In brief, the ADCHAM model^48,49^ was used to simulate the DMS experiments in the AURA chamber using the observed RH and temperature as input to the model. For this purpose, the model data was compared to results from HR-ToF-AMS to analyze the mass of the single components present in the aerosol particle mass. New particle formation was modeled using the atmospheric cluster dynamics code ACDC,^64^ considering neutral and ion-induced clustering of SA and NH_3_ molecules.^34^ ACDC was dynamically coupled to the aerosol dynamics module in ADCHAM using the methodology described in Roldin et al.^49^ The DMS gas-phase chemistry was primarily based on MCMv3.3.1.^65−67^

### 2.6 Quantum Chemical Calculations

Quantum chemical calculations were performed to obtain the molecular structures and thermochemistry of clusters consisting of SA, MSA, and ammonia (A). The Gaussian16 program^68^ (using G09 defaults) was employed to obtain the molecular cluster structures and calculate the harmonic vibrational frequencies. We used the ωB97X-D^69^ density functional as it has been shown to yield the lowest errors in the binding energies of atmospheric molecular clusters compared to higher-level CCSD(T) complete basis set estimates.^70−73^ The density functional theory calculations were performed using the 6-31++G(d,p) basis set, which has shown good agreement with basis sets of significantly larger sizes.^74,75^

Thermochemistry of the (SA)_1–4_(A)_1–4_ clusters was taken from the Atmospheric Cluster Database (ACDB).^76^ The cluster structures of the (MSA)_1–4_(A)_1–4_ clusters were taken from ref (77) and recalculated at the DLPNO-CCSD(T_0_)/aug-cc-pVTZ//ωB97X-D/6-31++G(d,p) level of theory. The (SA)_1–3_(MSA)_1–3_ and (SA)_*x*_(MSA)_*y*_(A)_1–4_, with *x* + *y* ≤ 4, were fully obtained in this work. The cluster structures were initially sampled using a CHARMM force field with the artificial bee colony (ABC) algorithm as implemented in the ABCluster program.^78,79^ The clusters were sampled using similar settings to those presented by Kubečka et al.^80^ We used the semiempirical PM7 method to reduce the number of relevant conformers based on the RMSD^81,82^ of the clusters using the ArbAlign program.^83^ Subsequently, the cluster structures and vibrational frequencies were calculated at the ωB97X-D/6-31++G(d,p) level of theory.

For all of the cluster structures, the binding energies were calculated using a high-level DLPNO-CCSD(T_0_)^84,85^ calculation with an aug-cc-pVTZ basis set using the ORCA program version 4.2.1.^86^ The auxiliary aug-cc-pVTZ/C and aug-cc-pVTZ/JK basis sets were used for density fitting and Coulomb/exchange fitting, respectively. We have previously demonstrated that this level of theory yields binding energy results in good agreement with higher-level coupled-cluster complete basis set estimates.^72,73^ All of the thermochemical parameters have been calculated at 298.15 K and 1 atm. All of the cluster structures and the associated thermochemical data are deposited in the Atmospheric Cluster Database (ACDB).^76^

## 3 Results and Discussion

A series of 11 experiments were conducted in the AURA chamber. Table 1 provides an overview of the experimental conditions as well as the aerosol mass yield, maximum aerosol mass, total particle number concentration, particle density, initial particle growth rate, and MSA to SO_4_^2–^ ratio.

### 3.1 Physical Properties

**Figure 1:**
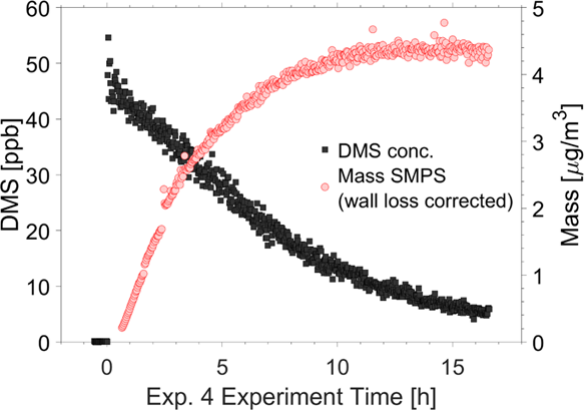
DMS concentration and wall-loss-corrected aerosol mass during Exp. 4, as measured from the combination of nano- and long-SMPS (using the density as retrieved from HR-ToF-AMS data). The time of DMS injection is *t* = 0.

Figure 1 presents an example of the decrease in DMS concentration and the associated increase in aerosol mass (based on SMPS data using the density as calculated from HR-ToF-AMS; see more information on this calculation below) during a typical experiment. The maximum particle mass in this example is approximately reached after 10 h. Corresponding results for the other experiments along with information about initial particle number concentration, T, RH, and ozone concentrations in the chamber are provided in Figures S2–S12 in the SI.

The aerosol mass yield (*Υ*) is defined as the mass of formed aerosol particles (*m*_a_) divided by the mass of reacted DMS (ΔDMS) and was calculated for the point in time where the maximum mass of aerosol particles (after wall loss correction) was reached^87^
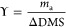
2

**Figure 2:**
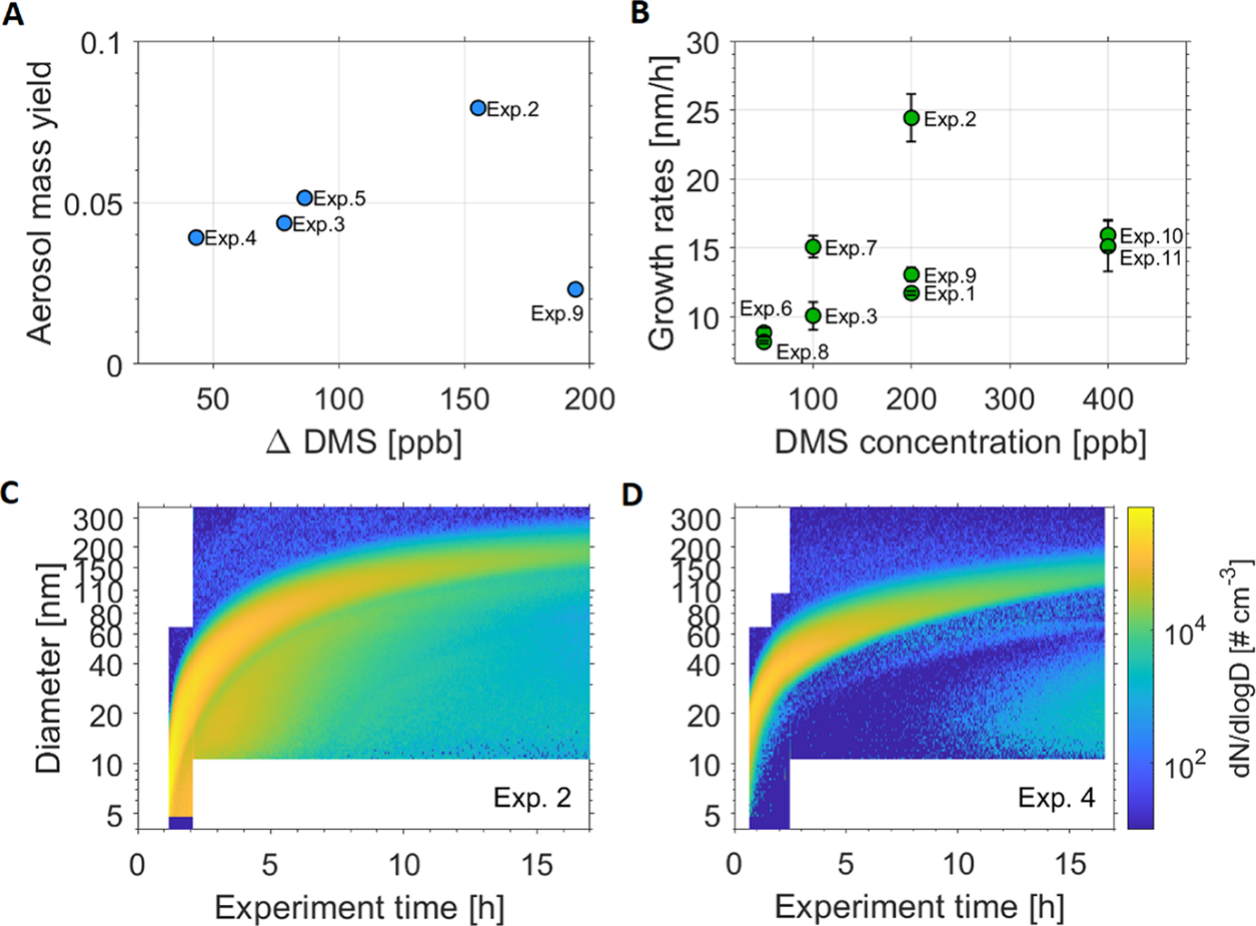
(A) Aerosol mass yields vs ΔDMS (see also Table 1). As the DMS concentration was not completely consumed when the maximum mass was reached, only data from experiments where the PTR was employed are shown. (B) Calculated growth rates (GR) using the maximum-concentration method^90,91^ for the size range from 10 to 20 nm. The error bars denote the uncertainty from the GR fit. The *x*-axis denotes the theoretical DMS concentrations injected into the chamber. (C and D) Size distribution plots recorded with nano- and long-SMPS systems for Exp. 2 (200 ppb DMS) and Exp. 4 (50 ppb DMS).

Figure 2A and Table 1 present the calculated aerosol mass yields for the different initial DMS concentrations in the AURA chamber. Particle mass concentrations are derived from SMPS measurements assuming spherical particles. The density of the particles was calculated based on the MSA and SO_4_^2–^ concentrations, as measured by the HR-ToF-AMS, considering that MSA has a density of 1.48 g/cm^3^^88^ and SA, measured as SO_4_^2–^ in the AMS, has a density of 1.84 g/cm^3^.^88^*m*_a_ was calculated using the densities listed in Table 1. During this study, DMS was not completely consumed when the maximum particle mass was reached, as can for example be seen in Figure 1, which is most probably related to insufficient levels of the oxidant. Thus, yield data can only be retrieved from experiments where the PTR-MS instrument was used to measure DMS concentrations. Figure 2 shows aerosol mass yields ranging from 0.02 to 0.08, with a slight trend toward increasing aerosol yield with increasing DMS consumption. The mass concentrations varied from 2.3 to 31.8 μg/m^3^, while the total number concentration of particles produced was relatively similar throughout all experiments ranging from 0.4 to 2.2 × 10^7^ #/cm^3^ (see Table 1).

A previous study by Chen and Jang^89^ investigated the aerosol mass yield from photo-oxidation experiments of DMS in the presence of 40–300 ppb of NO_*x*_. Although the DMS concentrations of Chen and Jang (116–170 ppb) are comparable to those in our study, their mass concentrations and mass yields strongly exceed our findings, reaching mass concentrations of 65.1–192 μg/m^3^ and yields of 0.536–0.753. Although Chen and Jang used a different method for estimating organic aerosol mass and an assumed aerosol particle density of 1.2, thus similar to that of DMS, the differences in mass yields are large. During the experiments in the current study, the NO_*x*_ values were always below 5 ppb; thus, it seems reasonable to conclude that NO_*x*_ has a large influence on the aerosol mass concentrations and mass yields. ADCHAM supports this conclusion as NO_*x*_ promotes the aerosol mass yield in the model by favoring CH_3_SO_3_ and ultimately SA and MSA production rather than SO_2_ formation. Besides, ADCHAM runs also showed that aerosol mass yields are sensitive to vapor wall loss rates and particularly dilution in the chamber (more details are presented in Section 3.2).

The temporal evolution of the two example particle size distributions (Exp. 2, 200 ppb DMS and Exp. 4, 50 ppb DMS), shown in Figure 2C,D, displays new particle formation with subsequent growth of the aerosol particles formed in the DMS photo-oxidation. After 10 h, particles in Exp. 2 grew to approximately 140 nm of particle mode diameter, while a smaller aerosol particle mode diameter of approximately 100 nm was measured in Exp. 4. Our results show a second growth mode below the main nucleation mode. This is evident for all DMS concentrations used in our experiments, thus for all experiments listed in Table 1. Growth rates (GRs) of fine aerosol particles with diameters between 10 and 20 nm were calculated from the aerosol size distributions using the maximum-concentration method^90,91^ and are displayed in Figure 2B and Table 1. Due to the experimental procedure, i.e., the subsequent measurements with first CPC, then nano-SMPS, and then long-SMPS, it was not possible to follow the growth of particle sizes below 10 nm. In Exp. 4 and 5, the size distribution measurement started later in time and hence the GR for this size range could not be determined. The measured GRs in our study ranged from 8 to 25 nm/h for DMS concentrations between 50 and 400 ppb. The results pointed toward slightly higher GRs with increasing DMS concentrations. Ambient measurements in marine, coastal areas found GRs between 1.8 and 20 nm/h for particles with diameters from 7 to 20 nm.^92,93^ Hence, the GRs observed from DMS oxidation here are in good agreement with findings in ambient air.

### 3.2 Chemical Composition and Model Simulations

**Figure 3:**
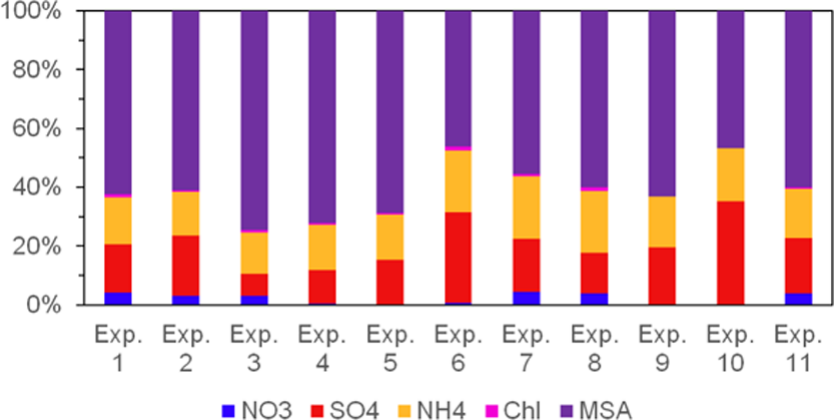
HR-ToF-AMS-measured particle composition shown as relative mass concentrationsof NO_3_^–^, SO_4_^2–^, NH_4_^+^, chloride, and MSA. Data are 10 min averages at the time of maximum aerosol mass.

**Figure 4:**
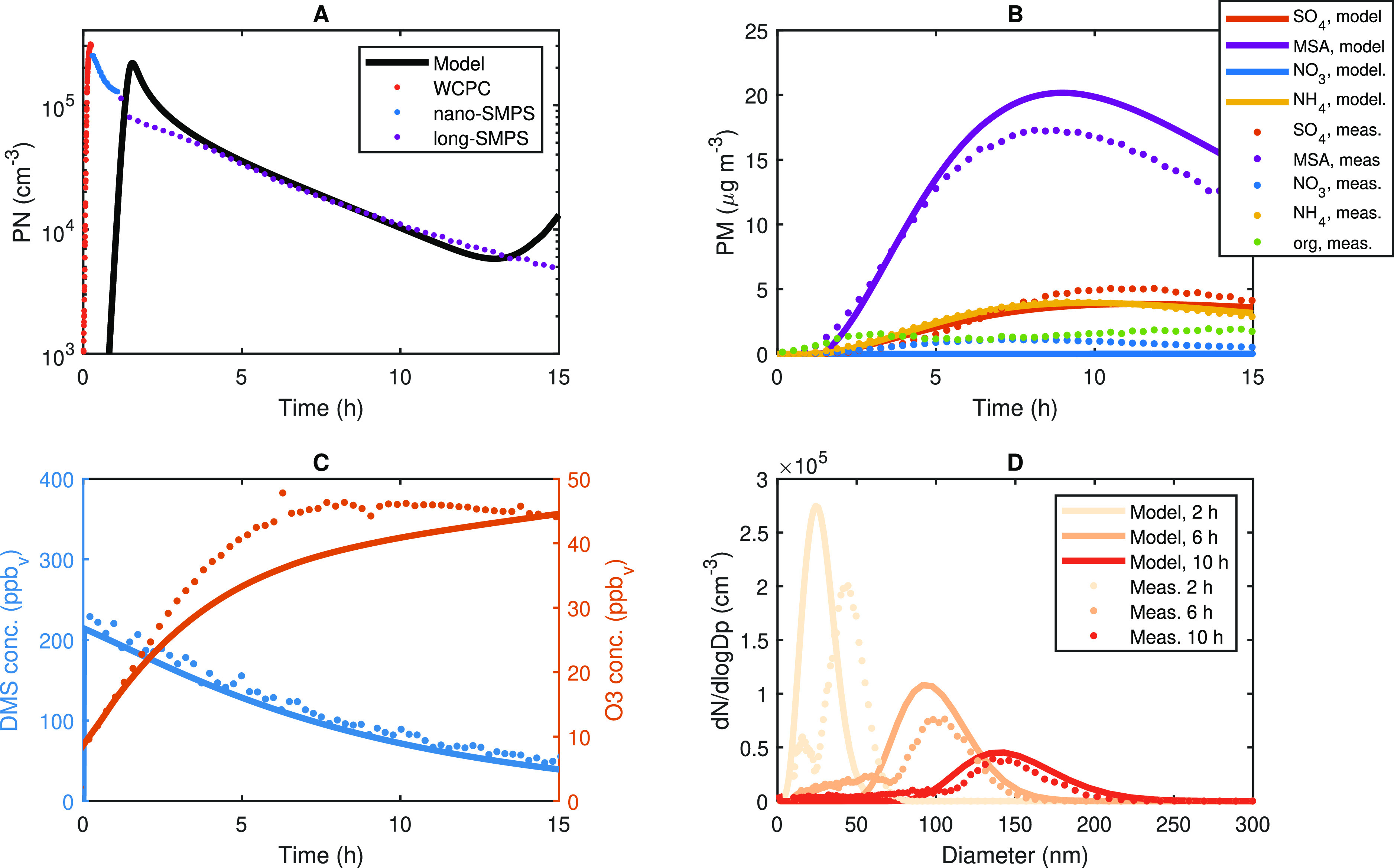
Measured and modeled results from Exp. 2. (A) Particle number concentration, (B) HR-ToF-AMS particle mass composition, (C) O_3_ (red) and DMS (blue) concentrations, and (D) SMPS particle size distribution.

The particle chemical composition for all experiments derived from AMS measurements is illustrated in Figure 3 as relative compositions of NH_4_^+^, NO_3_^–^, chloride, and the DMS oxidation products MSA and SA (measured as SO_4_^2–^) at the time of maximum aerosol mass in each experiment. Overall, the results show that OH oxidation of DMS resulted in a dominant contribution from MSA to particle mass compared to SO_4_^2–^ in all cases. Figure 4B shows an example of the temporal evolution of the chemical composition (from Exp. 2). Minor and variable contamination from NO_3_^–^ was observed, and low levels of chloride were found above the literature detection limit^60^ in Exp. 1–3, 7, and 11. In addition, low concentrations of constituents categorized as organic aerosol (OA) were observed (full results are shown in Figures S13 and S14 in the SI). The mass spectra of the measured OA include the ions CO^+^, CO_2_^+^, and CHO^+^ and a large number of minor contributions from fragments that could be unknown OA, which could include organic oxidation products from DMS other than MSA. CO_2_^+^ from oxidation of carbon residues on the AMS vaporizer^94^ was found to be negligible during calibrations. Part of the OA could be measurement artifacts, including MSA calibration uncertainty and indications of memory effects^95,96^ from high OA experiments few days prior to the experiments of this study. Comparing the absolute mass of OA across the experiments suggests that fairly constant contamination in the AMS data cannot be ruled out. It could not be determined whether the source of the contamination was the simulation chamber or deposits within the instrument. Assuming the measured OA to be an artifact or contamination implies that SO_4_^2–^ and MSA were the only measured particle-phase products at the time of maximum aerosol mass. The concentrations of OA have been omitted in Figure 3 not to obscure the comparison of relative compositions of low- and high-mass experiments. Table 1 presents measured MSA to SO_4_^2–^ ratios for all experiments, ranging from 1.3 to 10.4. There is a slight tendency toward higher MSA to SO_4_^2–^ ratios for smaller initial DMS concentrations. These are however also associated with a larger influence of potential organic contamination or memory effects in the AMS instrument as discussed above.

The Master Chemical Mechanism version 3.3.1 (MCMv3.3.1) gas-phase chemistry alone was not sufficient to describe the aerosol mass measured using the HR-ToF-AMS. At 293 K, the use of MCMv3.3.1 reactions underestimated the rate of SA and MSA production and hence the particle number (PN) and mass formation onset. By implementing the oxidation of MSIA to CH_3_SO_2_^97^ and isomerization of CH_3_SOO to CH_3_SO_2_,^98^ the overall rate of oxidation was increased. This ensured a decent representation of the initial rate of particle formation (Figure 4A,D) but overestimated the overall particle mass measured by the HR-ToF-AMS. To solve this, hydroperoxymethyl thioformate (HPMTF) formed through the newly discovered autoxidation pathway of DMS was implemented in the model.^8^ See Wollesen de Jonge et al.^50^ for details and the schematic of the major routes of formation of MSA and SA included in the model. Model results suggest that HPMTF remained in the gas phase, acting as a sulfur reservoir. Consequently, a large fraction of DMS did not form MSA and SA, which lowered the overall secondary aerosol mass yield obtained in the model to the concentration observed in the experiments (Figure 4B). MSA dominates the chemical composition in the secondary aerosol mass measured by the HR-ToF-AMS. The model indicates that a high HO_2_ gas-phase concentration (from the reaction of OH radicals and O_3_) favored the CH_3_SO_3_+HO_2_ reaction and hence MSA production.

ADCHAM was able to reproduce the DMS decay by OH radical-initiated oxidation (Figure 4C) and thus the OH radical concentration in the AURA chamber. The model reported an average OH concentration of 2.3 × 10^6^ cm^–3^ in accordance with measurement observations (SI). Model results for Exp. 3 and 4 are presented in the SI (Figures S15 and S16). The second particle mode observed in Figure 2C,D is not clearly reproduced by the ADCHAM model. The reason for the experimentally observed second mode should be further investigated in future studies. As indicated by the observations (Figure 2A), the modeled aerosol mass yields increase with increasing DMS concentrations (SI; Fig. S17). This can be explained by the generally increasing aerosol particle condensation sink with increasing DMS loading, which results in decreasing influence from the SA and MSA wall losses. When taking into account the wall losses of particles and vapors and the chamber dilution, the modeled aerosol mass yields fall close to the same value of 0.3 after 13 h, irrespective of the initial DMS loading (SI, Figure S17). It can be noted that all DMS oxidation pathways likely lead to the formation of SA and MSA, which without any losses would result in aerosol mass yields of 1.5. However, especially the OH oxidation pathways going via HPMTF and SO_2_ as intermediate products are too slow to contribute to any substantial aerosol particle mass formation in the AURA experiments.

Unexpected species measured by the HR-ToF-AMS included NH_4_^+^, NO_3_^–^, and various organic compounds. Aerosol NH_4_^+^ concentrations were captured well by the model, by incorporating an initial amount of ammonium on the Teflon walls, causing a gradual diffusion of NH_3_ into the chamber. NO_*x*_ contamination was also included in the model but did not explain the concentration of NO_3_^–^ measured by AMS. Regarding organics, it should be noted that they only contributed significantly to particle composition when the overall aerosol mass concentration was low (Figure S14). In fact, the mass concentrations in, for example, Exp. 6 are so low that they are close to the detection limit and thus the uncertainty in these measurements is high. The detection limit for organic mass measured with the AMS increases with an increased number of fragment ions fitted in the mass spectrum.^95^ With near 300 fitted organic fragment ions in these experiments, the detection limit for the organic fraction is estimated to be in the range of 0.25–0.5 μg m^–3^ as based on Drewnick et al.^95^ Another factor that might contribute to the high appearance of organic mass in low-mass laboratory experiments is the memory effect in the AMS instrument, as described by Pieber et al.^94^

### 3.3 Cluster Formation Mechanism

Using the ACDC code and the newly calculated quantum chemical data, we can gain explicit insight into the particle formation mechanism from OH oxidation of DMS. As the exact concentrations of the precursors are unknown, we assume that we form roughly 10^8^–10^9^ molecules cm^–3^ of SA and MSA from oxidation of DMS by OH radicals. These concentrations are a few orders of magnitude larger than expected in the ambient atmosphere but realistic considering the experimental conditions (50–200 ppb of DMS) and the modeling results. Ammonia (A) has been shown always to be present in simulation chambers,^30^ so we assume that we have approximately 1 ppb (range from 0.1 to 10 ppb) of ammonia in the chamber. Estimates of ammonia concentrations are based on data from Tange (56.352222°N, 9.5875°W; ∼50 km from Aarhus; http://ebas.nilu.no/) and knowledge that measurements in a nearby analytical laboratory routinely show typical concentrations of ammonia in the range of 1–10 ppb. To fully explore the mechanism, we allow all different types of cluster compositions, i.e., (SA)_4_(A)_4_, (MSA)_4_(A)_4_ and (SA)_*x*_(MSA)_*y*_(A)_4_, with *x* + *y* ≤ 4 clusters to grow outside the ACDC simulation box and to contribute to the new particle formation (NPF) rate. The absolute values of the ACDC simulation are shown in the SI at various concentration ratios of SA and MSA at 298.15 K.

For all of the studied SA to MSA ratios, the simulated NPF rates are very dependent on the mixing ratio of ammonia. At 0.1 ppb or below of ammonia, no particles are formed. Increasing the ammonia mixing ratio by a factor of 10 yields four orders of magnitude higher NPF rates, over the considered ammonia concentrations (0.1–10 ppb). These results are in good agreement with the modeling results (see Section 3.2), which also identified that the particle formation rate and secondary aerosol mass are much dependent on the ammonia concentration. In all cases, we see that the particle formation rate is low, which is consistent with the experimental observation that the secondary aerosol formation and growth from DMS oxidation by OH radicals is slow and many hours are required for the secondary aerosol mass to peak.

**Figure 5:**
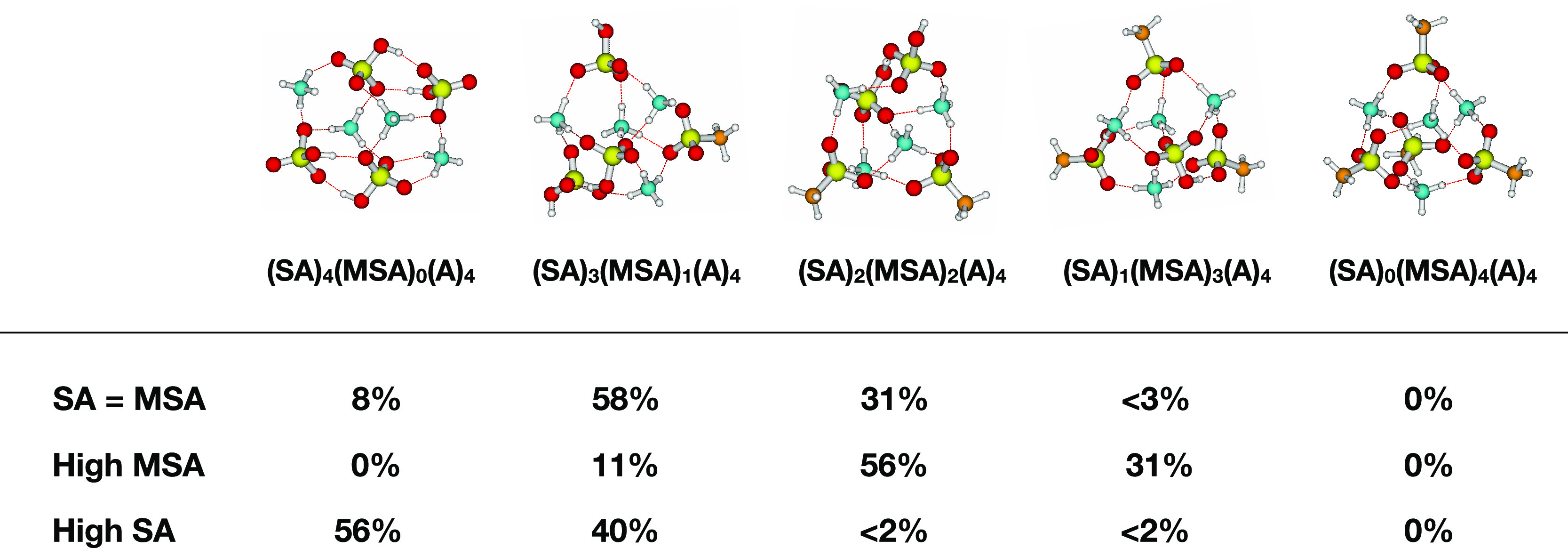
Molecular structures of the largest clusters considered, calculated at the DLPNO-CCSD(T_0_)/aug-cc-pVTZ//ωB97X-D/6-31++G(d,p) level of theory. The contribution (in %) of each of the clusters to the new particle formation rate at three different conditions (high SA, high MSA, and SA = MSA) at 1 ppb mixing ratio of ammonia is presented.

The formation mechanism of SA and MSA from the oxidation of DMS is inherently complex, and it remains unknown what the exact branching ratio is during the experiments. By simulating different ratios between SA and MSA, we can obtain insight into how this distribution affects the particle formation rate and mechanism. In the following, we will ignore cluster fluxes that contribute less than 5% to the particle formation rate. Figure 5 presents the molecular structures of the largest clusters studied and their individual contribution to the new particle formation rate under three different conditions: (1) high SA (10:1 SA/MSA ratio), (2) high MSA (1:10 SA/MSA ratio), and (3) SA = MSA (1:1 SA/MSA ratio). Here, 1 = 1 × 10^8^ molecules cm^–3^. In all cases, the ammonia concentration was fixed at 1 ppb.

With a 1:1 ratio of SA to MSA (1 × 10^8^ molecules cm^–3^) and 0.1 ppb of ammonia, 42% of the particles are formed from collisions with (SA)_3_(MSA)_1_(A)_4_ clusters, 29% of the particles are formed from collisions with (SA)_2_(MSA)_2_(A)_4_ clusters, 17% from (SA)_4_(A)_4_ clusters, and 6% from (SA)_1_(MSA)_3_(A)_4_ clusters. With increasing ammonia concentration, this distribution is perturbed, leading to a higher contribution from the (SA)_3_(MSA)_1_(A)_4_ cluster with 58 and 65% for 1.0 and 10 ppb of ammonia, respectively. This clearly indicates that newly formed particles from DMS oxidation will consist of different mixtures of both SA and MSA molecules when SA and MSA are present in equal concentrations.

Having a 10 times higher concentration of MSA compared to that of SA, the mechanism changes such that 63% of the particles are formed from collisions with (SA)_1_(MSA)_3_(A)_4_ clusters, with 31% contribution from (SA)_2_(MSA)_2_(A)_4_ clusters, at 0.1 ppb of ammonia. With increasing ammonia concentration, this mechanism is shifted toward 56 and 61% contributions from (SA)_2_(MSA)_2_(A)_4_ clusters at 1.0 and 10 ppb of ammonia, respectively.

With a 10 times higher concentration of SA compared to that of MSA, the particle formation mechanism is dominated by collisions with (SA)_4_(A)_4_ clusters (77%), with 16% contribution from (SA)_3_(MSA)_1_(A)_4_ clusters. This distribution is very similar for 1.0 ppb (56/40%) and 10 ppb (62/35%) of ammonia. In no cases do we see the formation of (MSA)_4_(A)_4_ clusters. Even by lowering SA to 1 × 10^6^ molecules cm^–3^ and increasing MSA to 1 × 10^10^ molecules cm^–3^ (yielding a 1:10 000 ratio), the growing clusters still have one sulfuric acid molecule present. These findings clearly show that the formed particles will consist of potentially different mixtures of SA and MSA molecules depending on the concentration ratio of the precursors. Most importantly, as these different particles consist of different acids, they might have very different growth properties and potentially also hygroscopicities. Thus, this distribution might explain the experimentally observed broad distribution of small (nucleation mode) particles as these could originate from the different particle compositions, which will have different growth rates. As the cluster formation rates are in all cases quite low, the particle formation mechanism in the ambient atmosphere involving DMS oxidation by OH radicals will most likely be driven by mixed SA/MSA clusters clustering with both amines and ammonia.

## 4 Conclusions

New particle formation and growth from gaseous dimethyl sulfide (DMS) oxidized by hydroxyl radicals were studied experimentally in the AURA chamber and complemented by simulations with the ADCHAM model and quantum chemical calculations. Experimental observations show that aerosol mass yields are generally low, that particle formation and growth rates are slow, and that the formed aerosols consist predominately of methanesulfonic acid, rather than sulfate. ADCHAM and experimental data could be reconciled by implementing new oxidation mechanisms to reactions described in the Master Chemical Mechanism version 3.3.1 used in the model as well as hydroperoxymethyl thioformate (HPMTF), recently found to be formed via an autoxidation pathway of DMS. ADCHAM suggests that HPMTF serves as a gaseous sulfur reservoir, leading to a large portion of DMS not forming MSA and SA (see also the companion paper by Wollesen de Jonge et al.^50^). Both the ADCHAM model and quantum chemical calculations reveal that new particle formation rates are strongly dependent on the mixing ratio of ammonia. Quantum chemical calculations also demonstrate that the formed particles can have different mixtures of MSA and SA, which is decisive for the particles’ properties such as their growth or water uptake potential.

In the marine atmosphere, there will always be some existing particles or cloud droplets resulting from long-range transport, sea spray emissions, local anthropogenic primary particle sources (e.g., shipping), or nucleation. These particles will be an adequate condensation sink for MSA and SA also without ammonia-mediated nucleation, and we expect that the secondary aerosol mass yields from DMS will not be limited by the presence of ammonia. In the chamber experiments discussed herein, ammonia becomes important for the secondary aerosol mass yields since we do not have initial seed particles and the chamber walls do contribute a substantial sink for both MSA and SA. However, such a major vapor deposition sink is not present in the atmosphere. In addition, recent observations at the coast of Antarctica and in the Arctic indicate that the observed new particle formation over the open ocean is driven by ammonia-SA clustering.^99,100^ Thus, although the ammonia concentrations in our chamber experiments are orders of magnitude higher than in polar marine environments, we expect that it is the same type of nucleation mechanism that dominates new particle formation.

## Supporting Information Available

Experimental estimations of OH radicals in the chamber, details on the MSA calibration of the HR-ToF-AMS, general information on each experiment together with more details on the chemical composition and model parameters, and comparison between experimental and modeled data and modeled aerosol mass yield data and ACDC simulations (PDF)
