# Supplementary material for: New Particle Formation and Growth from Dimethyl Sulfide Oxidation by Hydroxyl Radicals
Source: ACS Earth Space Chem. 2021 Mar 25;5(4):801–11. doi: 10.1021/acsearthspacechem.0c00333 (PMC8054244; doi:10.1021/acsearthspacechem.0c00333)
Supplement: Supplementary file 1 — sp0c00333_si_001.pdf [file sp0c00333_si_001.pdf]

Supporting Information:

New Particle Formation and Growth from Dimethyl Sulfide Oxidation by Hydroxyl Radicals

Bernadette Rosati *[a,b], Sigurd Christiansen[a], Robin Wollesen de Jonge[a], Pontus Roldin[c], Mads Mørk Jensen[a], Kai Wang[a], Shamjad P. Moosakutty[a,d], Ditte Thomsen[a], Camilla Solomonsen[a], Noora Hyttinen[e,f], Jonas Elm[a], Anders Feilberg[g], Marianne Glasius[a], and Merete Bilde†[a]

[a]Department of Chemistry, Aarhus University, DK-8000 Aarhus C, Denmark
[b]Faculty of Physics, University of Vienna, AT-1090 Vienna, Austria
[c]Division of Nuclear Physics, Lund University, P.O. Box 118, Lund, Sweden
[d]Clean Combustion Research Center, King Abdullah University of Science and Technology, Thuwal, Saudi Arabia
[e]Nano and Molecular Systems Research Unit, University of Oulu, P.O. Box 3000, Oulu, Finland
[f]Department of Applied Physics, University of Eastern Finland, P.O. Box 1627, Kuopio, Finland
[g]Department of Engineering, Aarhus University, Finlandsgade 12, Aarhus, Denmark

*bernadette.rosati@chem.au.dk
†bilde@chem.au.dk

- OH radical estimations in experiments in AURA chamber: Figure S1, Table S1
- Non-default entries of the HR fragmentation table applied to account for MSA: Table S2
- General data for all 11 experiments: Figures S2 - S12
- Aerosol particle chemical composition: Figures S13 - S14
- Specific model parameters used for the simulations of experiments 2,3 and 4
- Comparison between measured and modeled results: Figures S15 - S16
- Modeled aerosol mass yields: Figure S17
- ACDC Simulations: Table S4
- Bibliography

OH radical estimation:

The concentration of OH radicals was estimated from supplementing experiments where 1-butanol was oxidized by OH radicals. 1-butanol (purity $> 99\%$, Sigma Aldrich) was introduced into the AURA chamber and under the assumption that the 1-butanol decay is solely due to reactions with OH radicals in the chamber, the time dependent OH concentration can be calculated assuming pseudo-first order conditions as for example proposed by Sang et al.[(2)]:

$$[OH] = \frac{ln\frac{B(t)}{B_0}}{k \cdot t}, \tag{0.1}$$

where $B(t)$ and B_0 denote the butanol concentration at a certain time (t) during the experiment and at time zero and k is the rate constant for the reaction of OH with 1-butanol. Figure S1 illustrates the loss of 1-butanol with time for three experiments with high and low H_2O_2 concentrations (1500 vs. 418 μL) and different sets of lamps in the AURA chamber respectively. Table S1 presents the slopes of linear least squares fit to the experimental data and derived OH concentrations. The results demonstrate that the OH concentrations in the chamber were comparable when using the high and low H_2O_2 concentrations and slightly higher when using a completely new set of UV lamps. These OH concentrations are in the range of the tropospheric concentrations of 1.50E+06 molecules/cm^3 [(1)].

Table S1: 1-butanol experiments in AURA: Fit results from decay rates illustrated in Fig. S1. k_{exp} denotes the slope of a linear least squares fit to the experimental data ($\frac{ln\frac{B(t)}{B_0}}{t}$). The OH concentration was derived using a rate constant for the reaction of OH radicals with 1-butanol of $8.72 \cdot 10^{-12}$ cm^3 molecules^{-1}s^{-1} at T=293K[(4)] (Eq. 0.1). The RH in all experiments was 0%.

Date	H_2O_2 [μL]	k_{exp} [s^{-1}]	OH [molecules/cm^3]
26.03.2019	418	-4.09E-05	4.68E+06
02.04.2019	1500	-4.13E-05	4.73E+06
12.04.2019	418	-4.98E-05	5.71E+06

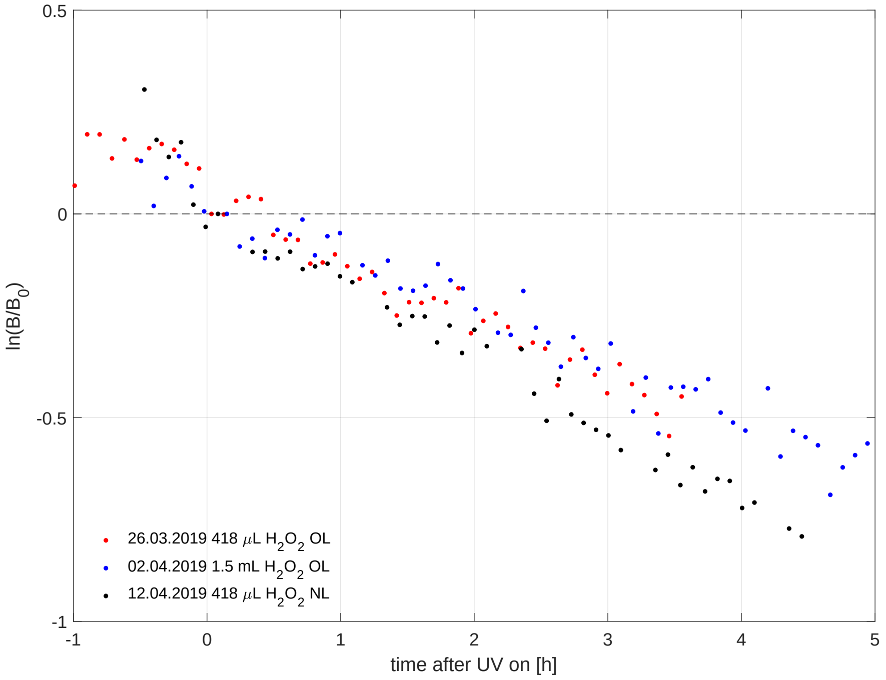

Figure S1: 1-butanol decrease rates at different oxidant and lamp conditions as employed during the campaign. OL: old lamps; NL: new lamps;

AMS analysis:

Table S2: Non-default entries of the HR fragmentation table applied to account for MSA. Isotope entries are not shown.

Ion	Sulfate	Organic	MSA
C		C-MSA[C]	0.068*MSA[CH3SO2]
CH		CH-MSA[CH]	0.165*MSA[CH3SO2]
CH2		CH2-MSA[CH2]	0.602*MSA[CH3SO2]
CH3		CH3-MSA[CH3]	4.444*MSA[CH3SO2]
CH4		CH4-MSA[CH4]	0.066*MSA[CH3SO2]
CHO		CHO-MSA[CHO]	0.585*MSA[CH3SO2]
CH2O		CH2O-MSA[CH2O]	0.183*MSA[CH3SO2]
CH3O		CH3O-MSA[CH3O]	0.870*MSA[CH3SO2]
CH4O		CH4O-MSA[CH4O]	0.154*MSA[CH3SO2]
CHS		CHS-MSA[CHS]	0.601*MSA[CH3SO2]
CH2S		CH2S-MSA[CH2S]	0.253*MSA[CH3SO2]
CH3S		CH3S-MSA[CH3S]	0.195*MSA[CH3SO2]
SO	SO-MSA[SO]		1.005*MSA[CH3SO2]
HSO	HSO-MSA[HSO]		0.090*MSA[CH3SO2]
H2SO	H2SO-MSA[H2SO]		0.020*MSA[CH3SO2]
CSO		CSO-MSA[CSO]	0.008*MSA[CH3SO2]
CHSO		CHSO-MSA[CHSO]	0.002*MSA[CH3SO2]
CH2SO		CH2SO-MSA[CH2SO]	0.055*MSA[CH3SO2]
CH3SO		CH3SO-MSA[CH3SO]	0.114*MSA[CH3SO2]
SO2	SO2-MSA[SO2]		0.709*MSA[CH3SO2]
HSO2	HSO2-MSA[HSO2]		0.610*MSA[CH3SO2]
H2SO2	H2SO2-MSA[H2SO2]		0.056*MSA[CH3SO2]
CH2SO2		CH2SO2-MSA[CH2SO2]	0.336*MSA[CH3SO2]
CH3SO2			CH3SO2
SO3	SO3-MSA[SO3]		0.015*MSA[CH3SO2]
HSO3	HSO3-MSA[HSO3]		0.501*MSA[CH3SO2]
CH4SO3		CH4SO3-MSA[CH4SO3]	0.505*MSA[CH3SO2]

General data during the 11 experiments:

All mass concentrations are based on SMPS data, were corrected for wall-losses and calculated assuming a density of 1 g/cm^3. Notice that in Figure 1 in the main manuscript the density obtained from AMS data was used.

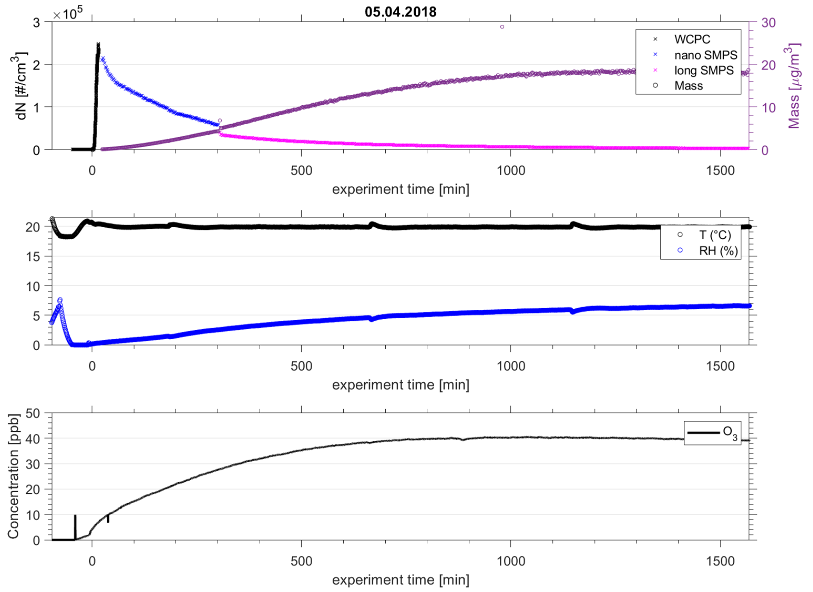

Figure S2: Exp 1: SMPS number concentrations and wall-loss corrected mass, recorded RH and T as well as O_3 concentrations.

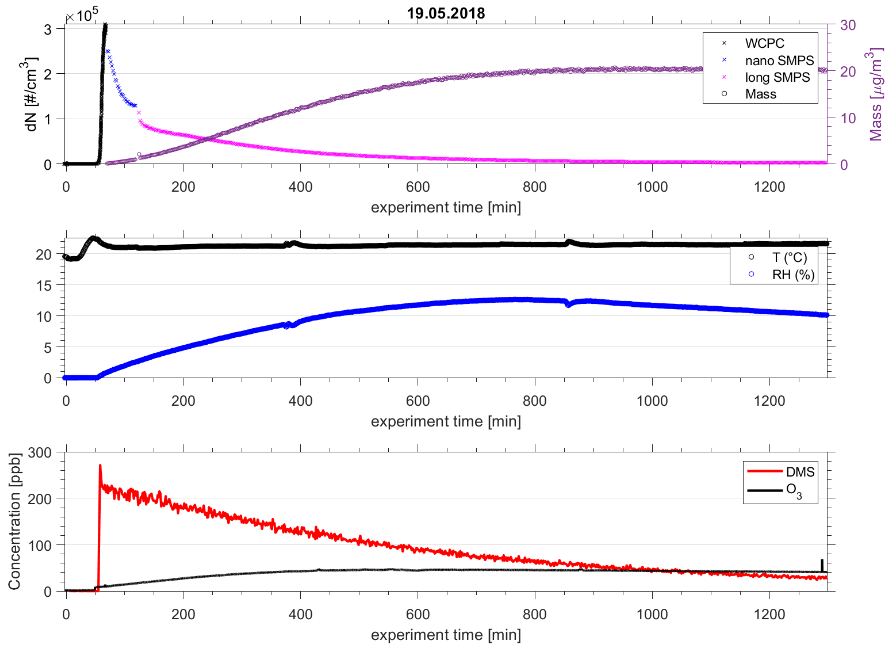

Figure S3: Exp 2: SMPS number concentrations and wall-loss corrected mass, recorded RH and T as well as DMS and O_3 concentrations.

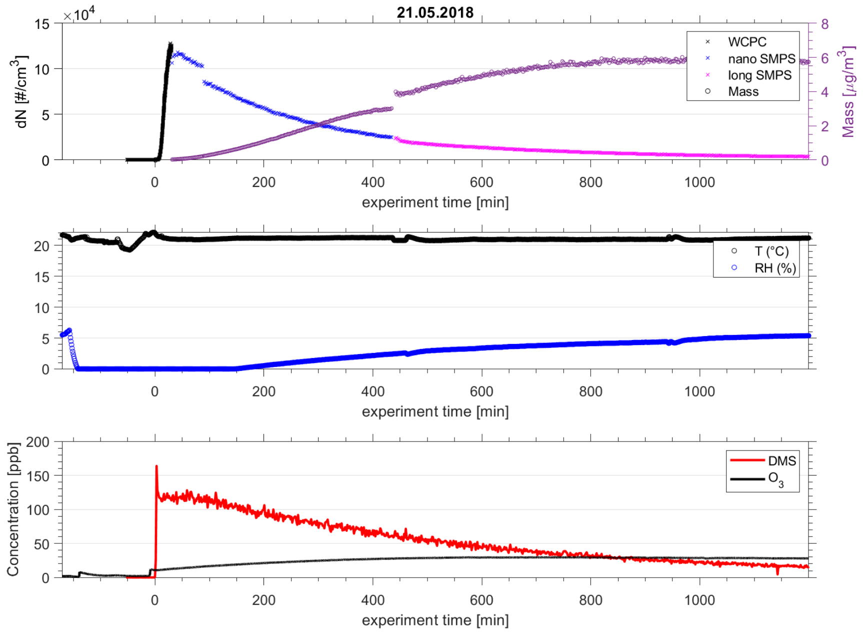

Figure S4: Exp 3: SMPS number concentrations and wall-loss corrected mass, recorded RH and T as well as DMS and O_3 concentrations.

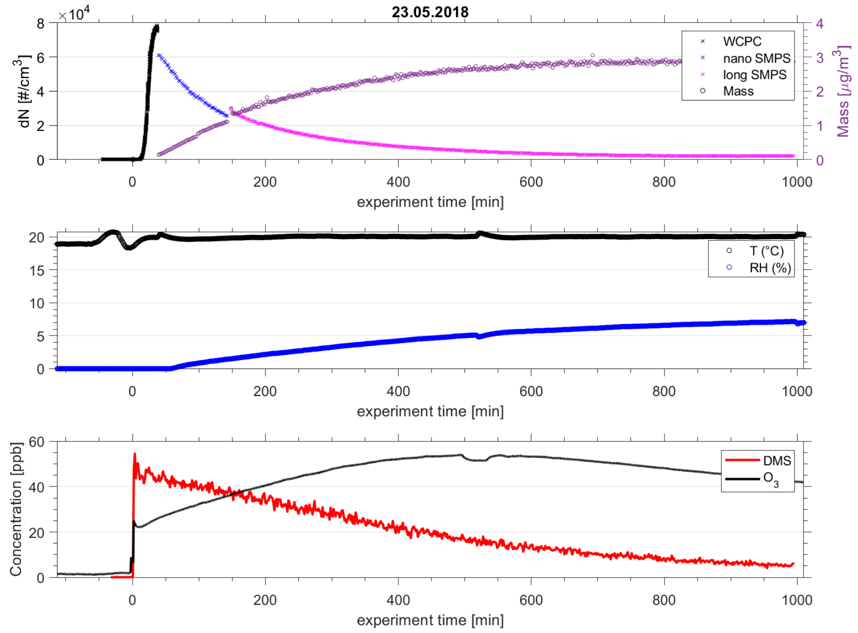

Figure S5: Exp 4: SMPS number concentrations and wall-loss corrected mass, recorded RH and T as well as DMS and O_3 concentrations.

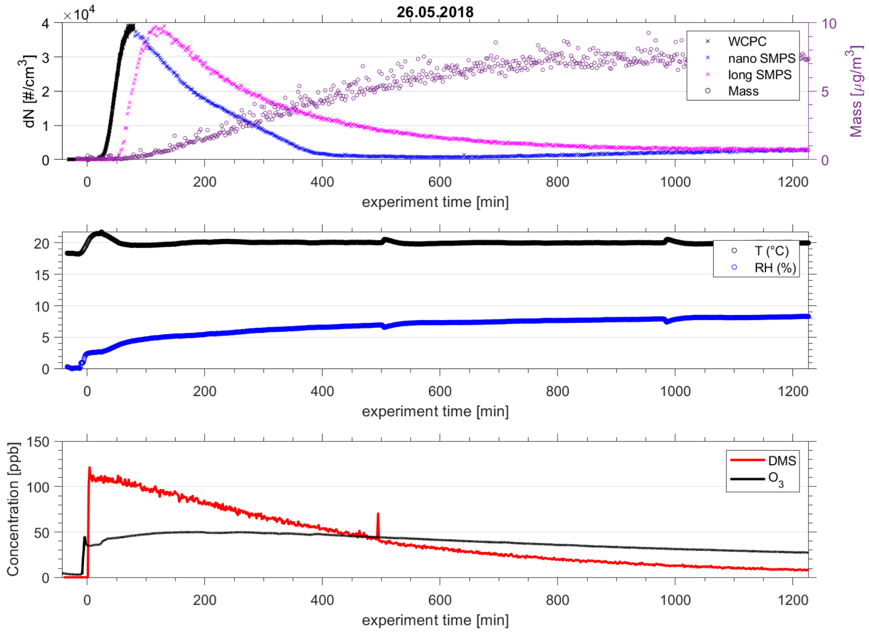

Figure S6: Exp 5: SMPS number concentrations and wall-loss corrected mass, recorded RH and T as well as DMS and O_3 concentrations.

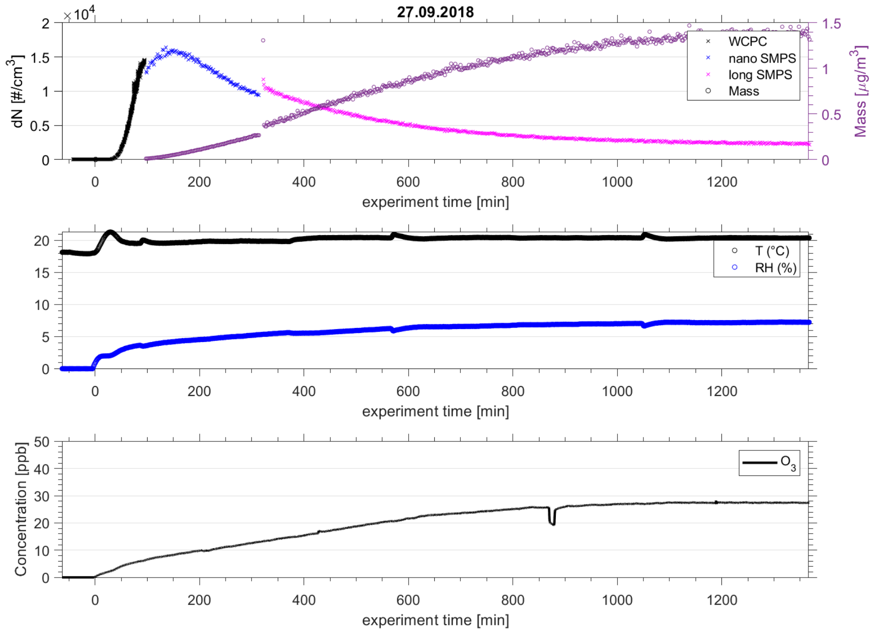

Figure S7: Exp 6: SMPS number concentrations and wall-loss corrected mass, recorded RH and T as well as DMS and O_3 concentrations.

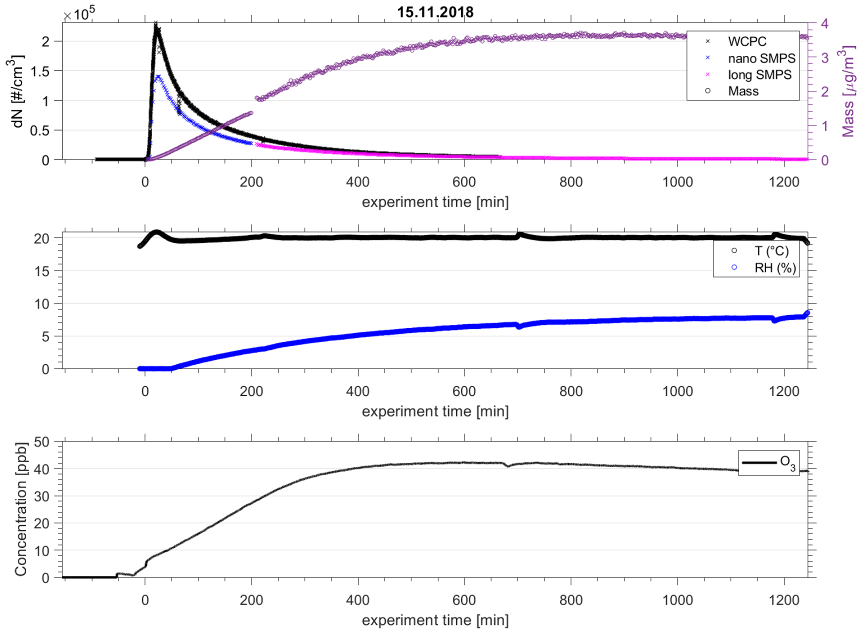

Figure S8: Exp 7: SMPS number concentrations and wall-loss corrected mass, recorded RH and T as well as DMS and O_3 concentrations.

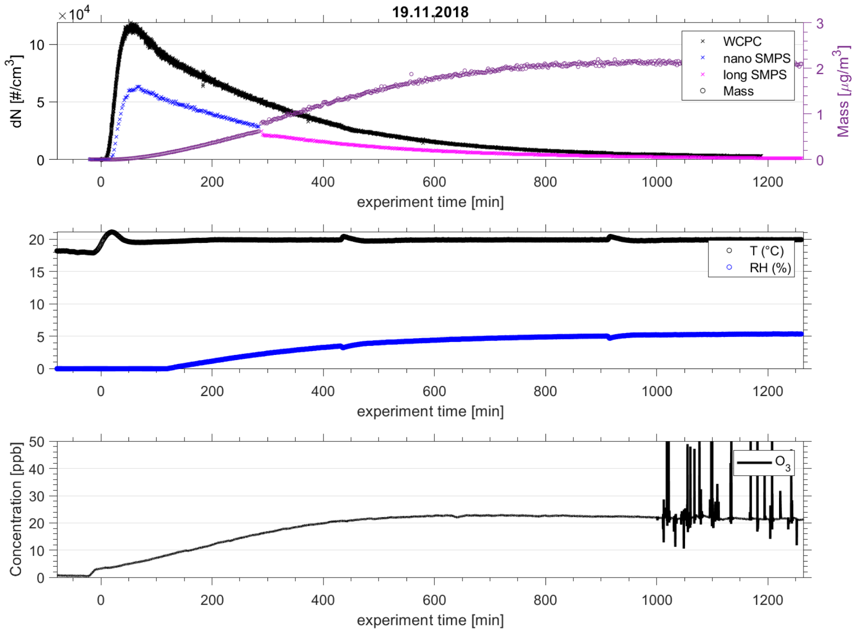

Figure S9: Exp 8: SMPS number concentrations and wall-loss corrected mass, recorded RH and T as well as DMS and O_3 concentrations.

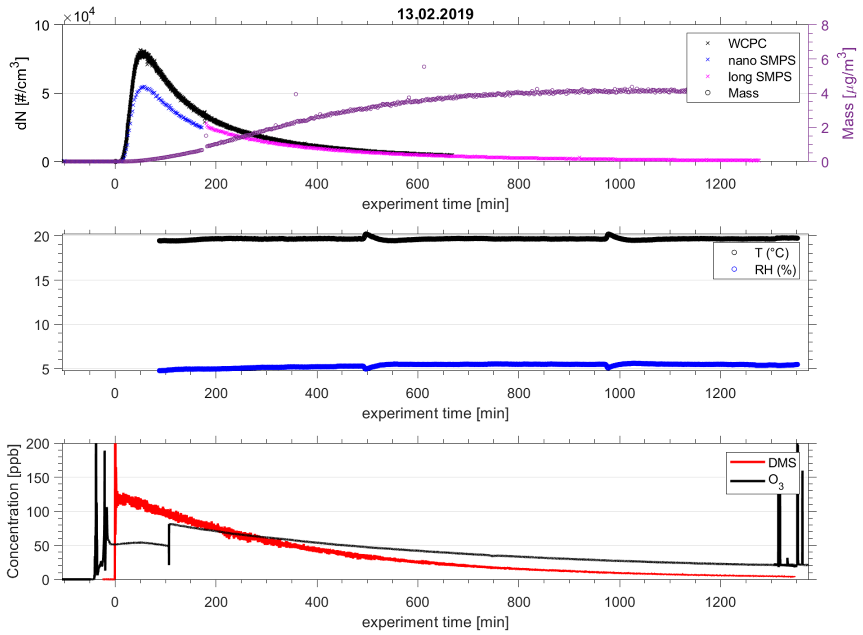

Figure S10: Exp 9: SMPS number concentrations and wall-loss corrected mass, recorded RH and T as well as DMS and O_3 concentrations.

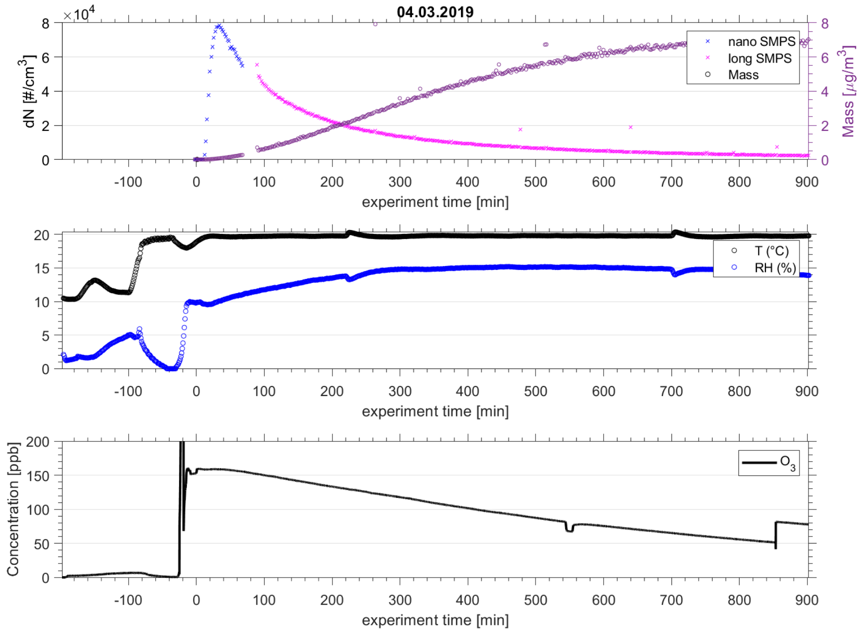

Figure S11: Exp 10: SMPS number concentrations and wall-loss corrected mass, recorded RH and T as well as DMS and O_3 concentrations.

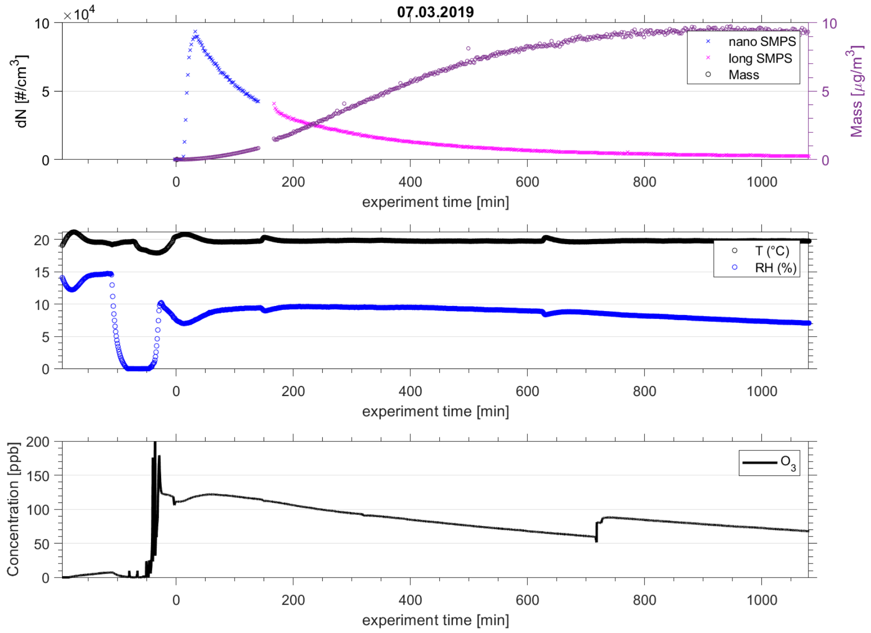

Figure S12: Exp 11: SMPS number concentrations and wall-loss corrected mass, recorded RH and T as well as DMS and O_3 concentrations.

Aerosol particle chemical composition:

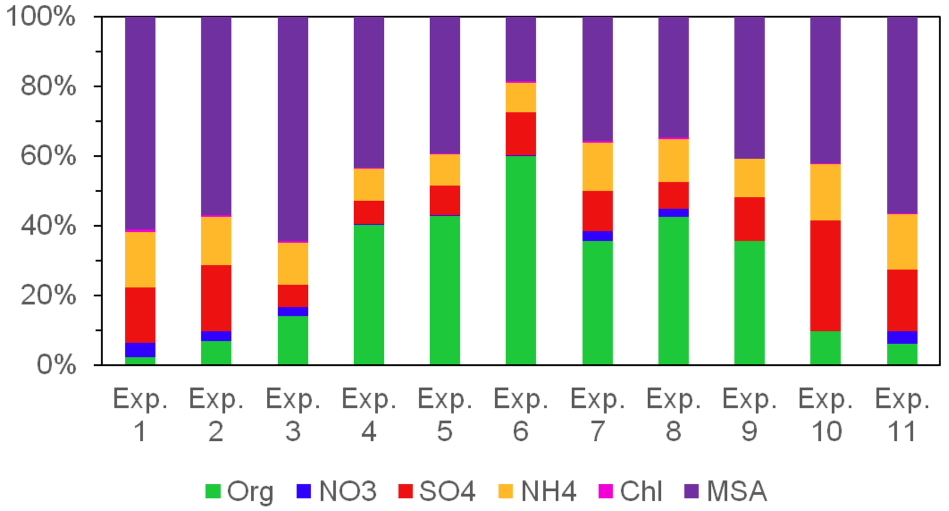

Figure S13: Particle composition shown as measured relative AMS mass concentrations. Data is an 10 min average around the time of maximum aerosol mass.

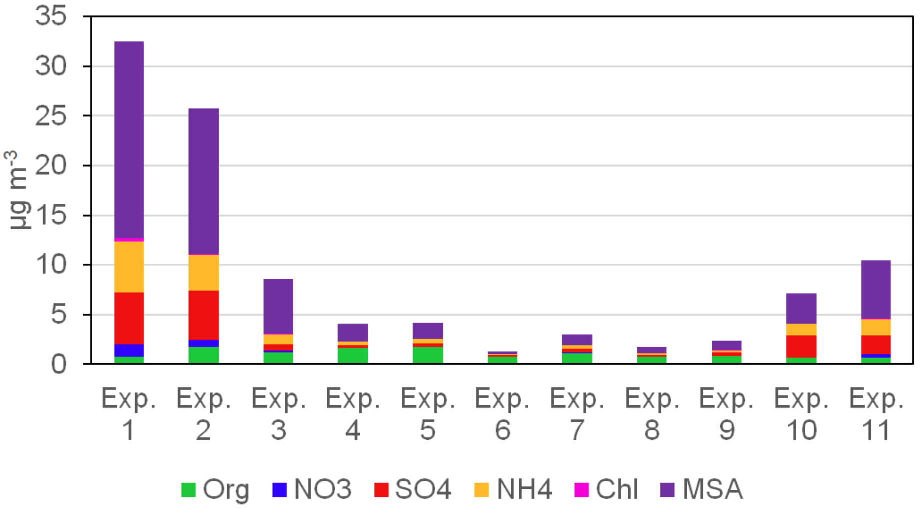

Figure S14: Particle composition shown as measured AMS mass concentrations. Data is an 10 min average around the time of maximum aerosol mass.

Specific model parameters used for the simulations of experiments 2,3 and 4:

Table S3 gives the values of key unknown model fitting parameter which influence the modeled wall losses of particles and gases and the evaporation of ammonia from the chamber walls. u^* and E_{field} are the friction velocity and the electric field strength respectively. These two model fitting parameters influence the wall losses of non-charged and charged particles respectively. k_e and LWC_{wall} are the coefficient of eddy diffusion and the effective liquid water concentration on the chamber walls respectively. k_e represent how fast the gas molecules are transported from the gas-phase to the chamber walls. LWC_{wall} mainly influence how much of the water soluble intermediate DMS oxidation products that are dissolved and oxidized in the water film on the walls. pH_{wall} and $[NH_4{}^+{}_{wall}]$ represents the pH and ammonium ion concentration in the liquid water film on the chamber walls. Both parameters influence how much ammonia which is released from the chamber walls. For a detailed description of the modeled wall losses of particles and gases the reader is referred to Wollesen de Jonge et al.[(3)]. The modeled PM was constrained based on the AMS observations.

Table S3: Key ADCHAM model parameter values which influence the gas and particle wall losses and the ammonia evaporation from the chamber walls

Exp.	u^* (m/s)	k_e (s^{-1})	E_{field} (V/cm)	LWC_{wall} (g/m^3)	pH_{wall}	$[NH_4{}^+{}_{wall}]$ (mol/(kg))
2	0.02	0.1	10	$3.5 \cdot 10^{-7}$	1	10
3	0.02	0.1	10	$3.5 \cdot 10^{-7}$	1	10
4	0.02	0.1	5	$3.5 \cdot 10^{-7}$	1	10

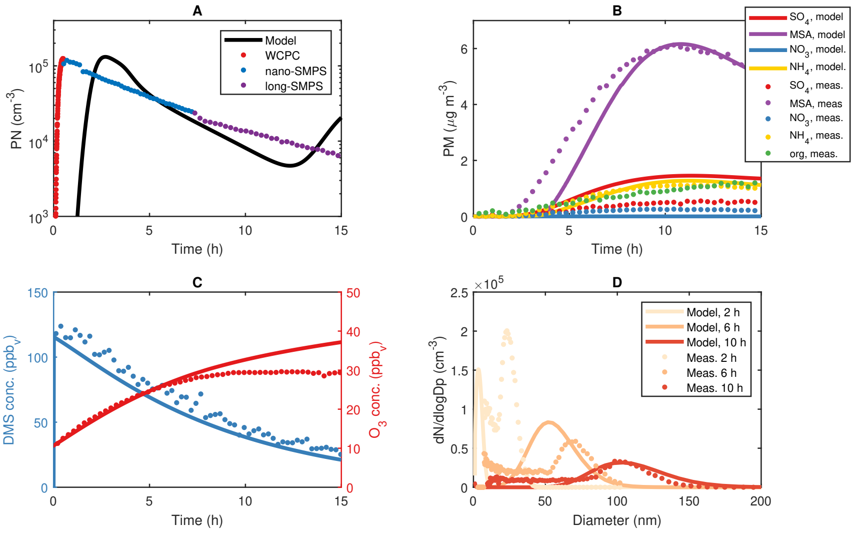

Figure S15: Measured and modeled results from Exp. 3. Panel **A** depicts the particle number concentration, **B** the HR-ToF-AMS particle mass composition, **C** the O_3 and DMS concentrations and **D** the particle size distribution.

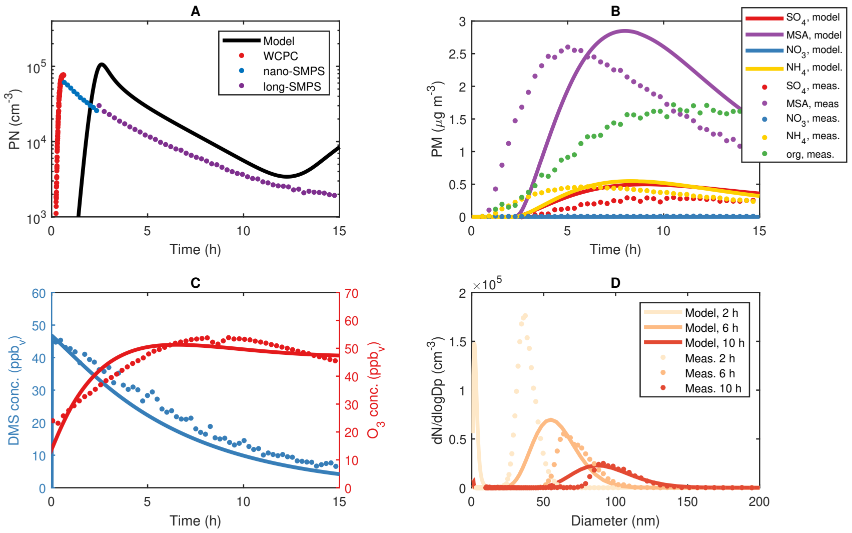

Figure S16: Measured and modeled results from Exp. 4. Panel **A** depicts the particle number concentration, **B** the HR-ToF-AMS particle mass composition, **C** the O_3 and DMS concentrations and **D** the particle size distribution.

Modeled aerosol mass yields with and without wall losses and chamber dilution:

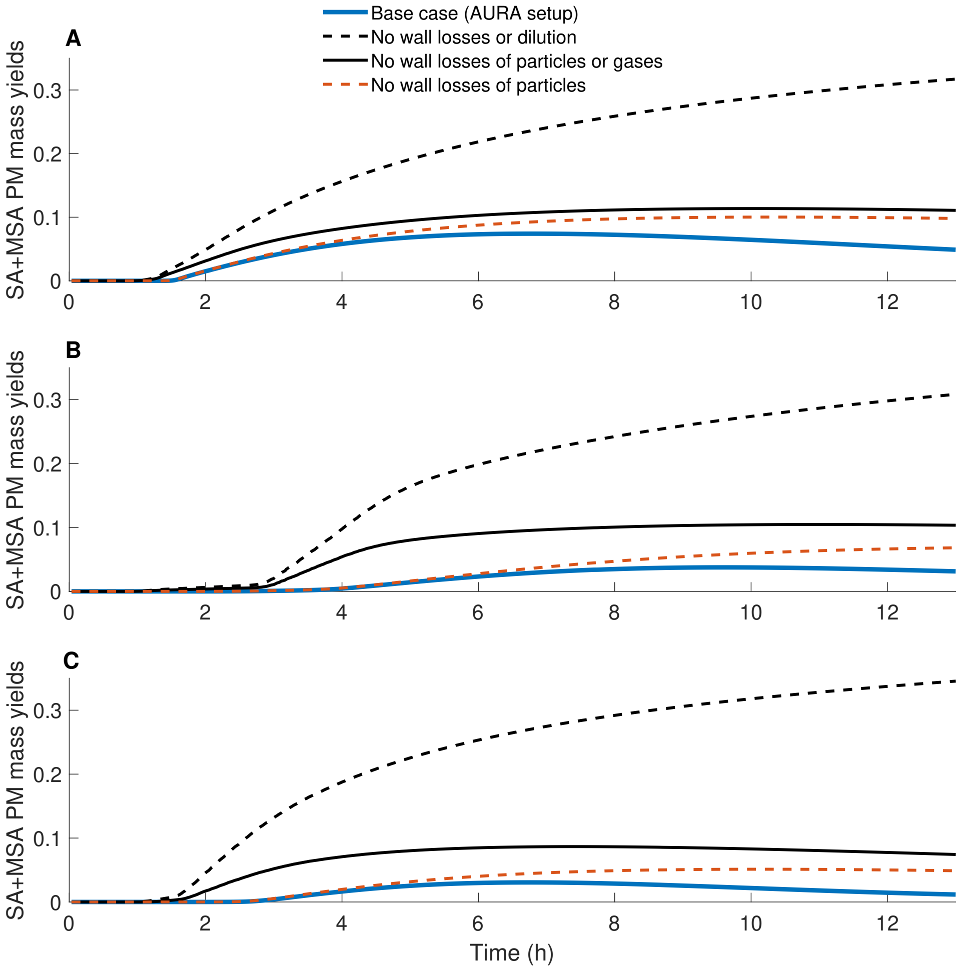

Figure S17: Modeled aerosol mass yields from **A** Exp. 2, **B** Exp. 3 and **C** Exp. 4. The model results were generated by running the model with the default AURA setup, without chamber dilution and any wall losses of particles and gases, without wall losses of particles and gases and without wall losses of particles.

ACDC Simulations

Table S4: Influence of the concentration ratio of SA and MSA (with 1 = $1{\cdot}10^8$ molecules cm^{-3}) on the simulated new particle formation rate.

SA:MSA	A	NPF rate
1:1	0.1 ppb	$1.6{\cdot}10^{-12}$
1:1	1.0 ppb	$8.6{\cdot}10^{-8}$
1:1	10 ppb	$4.8{\cdot}10^{-4}$
1:10	0.1 ppb	$1.8{\cdot}10^{-10}$
1:10	1.0 ppb	$4.7{\cdot}10^{-6}$
1:10	10 ppb	$1.8{\cdot}10^{-2}$
10:1	0.1 ppb	$4.2{\cdot}10^{-9}$
10:1	1.0 ppb	$1.2{\cdot}10^{-4}$
10:1	10 ppb	$8.6{\cdot}10^{-1}$
10:10	0.1 ppb	$2.1{\cdot}10^{-8}$
10:10	1.0 ppb	$8.7{\cdot}10^{-4}$
10:10	10 ppb	4.77

Bibliography

[1] J. H. Seinfeld and S. N. Pandis. *Atmospheric Chemistry and Physics: From Air Pollution to Climate Change*. John Wiley & Sons, Hoboken, NJ, 3rd edition, 2016. ISBN 978-1-118-94740-1.

[2] M. Song, C. Zhang, H. Wu, Y. Mu, Z. Ma, Y. Zhang, J. Liu, and X. Li. The influence of oh concentration on soa formation from isoprene photooxidation. *Science of The Total Environment*, 650:951 – 957, 2019. ISSN 0048-9697. doi: https://doi.org/10.1016/j.scitotenv.2018.09.084. URL http://www.sciencedirect.com/science/article/pii/S0048969718335216.

[3] R. Wollesen de Jonge, J. Elm, B. Rosati, S. Christiansen, N. Hyttinen, D. Lüdemann, M. Bilde, and P. Roldin. Secondary aerosol formation from dimethyl sulfide – improved mechanistic understanding based on smog chamber experiments and modelling. *Atmospheric Chemistry and Physics Discussions*, 2021:1–33, 2021. doi: 10.5194/acp-2020-1324. URL https://acp.copernicus.org/preprints/acp-2020-1324/.

[4] M. Yujing and A. Mellouki. Temperature dependence for the rate constants of the reaction of oh radicals with selected alcohols. *Chemical Physics Letters*, 333(1):63 – 68, 2001. ISSN 0009-2614. doi: https://doi.org/10.1016/S0009-2614(00)01346-4. URL http://www.sciencedirect.com/science/article/pii/S0009261400013464.
